# Interstitial washdown and vascular albumin refill during fluid infusion: novel kinetic analysis from three clinical trials

**DOI:** 10.1186/s40635-021-00407-6

**Published:** 2021-08-27

**Authors:** Robert G. Hahn, Randal O. Dull

**Affiliations:** 1 Karolinska Institute at Danderyds Hospital (KIDS), Stockholm, Sweden; 2 Research Unit, Södertälje Hospital, 152 86, Södertälje, Sweden; 3 Department of Anesthesiology, University of Arizona College of Medicine, Tucson, AZ USA; 4 Department of Pathology, University of Arizona College of Medicine, Tucson, AZ USA; 5 Department of Surgery, University of Arizona College of Medicine, Tucson, AZ USA

**Keywords:** Body water, Physiology, Crystalloid solutions, Pharmacokinetics, Extracellular space, Physiology, Pharmacokinetics, Saline solution, Isotonic

## Abstract

**Background and aims:**

Increased capillary filtration may paradoxically accelerate vascular refill of both fluid and albumin from the interstitial space, which is claimed to be edema-preventing. We characterized this proposed mechanism, called “interstitial washdown”, by kinetic analyses of the hemodilution induced by intravenous infusion of crystalloid fluid during 3 distinct physiological states.

**Methods:**

Greater plasma dilution of hemoglobin as compared to albumin during fluid therapy indicated recruitment of albumin, which was compared to the flow of interstitial fluid to the plasma as indicated by population volume kinetic analysis. Data for the comparison were derived from 24 infusions of crystalloid fluid in conscious volunteers, 30 in anesthetized patients, and 31 in patients with ketoacidosis from hyperglycemia.

**Results:**

“Interstitial washdown” increased the plasma albumin concentration by between 0.3 and 1.0 g/L in the three series of infusions. The initial albumin concentration in the interstitial fluid returning to the plasma was estimated to between 22 g/L and 29 g/L, which decreased to an average of 50–75% lower during the subsequent 2–3 h. Kinetic simulations show that pronounced washdown was associated with increased capillary filtration (high *k*_12_) and, in conscious subjects, with greater plasma and interstitial volume expansion and restricted urine flow. During anesthesia, the main effect was an increase in the non-exchangeable fluid volume (“third-spacing”).

**Conclusions:**

Crystalloid fluid accelerates lymphatic flow that moderately increases plasma albumin, but more clearly helps to maintain the intravascular volume. This “interstitial washdown” mechanism becomes exhausted after a few hours.

## Introduction

Current evidence suggests that distribution and kinetics of crystalloid fluid differ greatly between the awake state, general anesthesia, and in pathological metabolic syndromes [1].

Unresolved issues remain with regard to the turnover of crystalloid fluid in the human body that may serve to explain such differences. One is the large increase in non-exchangeable volume (e.g., the “third space”) that limits intravascular volume overload during general anesthesia [2]. Second is the poorly studied mechanism of “interstitial washdown” originally suggested by the eminent physiologist Arthur Guyton to counteract peripheral edema when capillary filtration is increased [3]. A task for clinicians is to characterize and quantify these and other fundamental physiological processes to form a rationale to improve fluid resuscitation strategies.

The present study explores this “interstitial washdown”, which implies that interstitial albumin is transferred to the plasma either by acceleration of the lymphatic flow or by some other mechanism that recruits albumin. The process may have similarities to the translocation of fluid following hemorrhage [4] and the change in body position from standing to supine [5], which are well studied compensatory mechanisms that restore plasma volume in response to absolute or relative hypovolemia. Lymphatic flow increases promptly in response to intravenous volume loading in the dog [6], but whether the induced flow is sufficient to prevent edema during crystalloid fluid therapy in humans is unknown. In fact, “interstitial washdown” has never been quantified.

How “interstitial washdown” might prolong the plasma volume expansion at the expense of interstitial edema during crystalloid fluid therapy can be summarized as follows: infusion of crystalloid is known to greatly increase the rate of capillary leakage of fluid from the plasma to the interstitial space [7]. After the infusion ends, volume is returned to the plasma to match the stimulated renal excretion. The returned fluid could, in whole or in part, consist of lymph, which contains both albumin and globulins in approximately half as high concentration as the plasma [8, 9]. Although the plasma constantly leaks small amounts of albumin, an accelerated lymphatic flow could return more protein than is lost, resulting in intravascular protein enrichment.

In the present analysis, data from three series of human trials (volunteers, anesthesia, and ketoacidosis patients) were used to quantify the plasma protein enrichment and its consequences on fluid distribution during crystalloid fluid therapy in humans.

The method of study was volume kinetics, which resembles drug pharmacokinetics, but uses the hemodilution induced by an infusion fluid instead of a drug concentration as the dependent variable [7]. Hemodilution can be calculated based on either hemoglobin (Hb) or albumin, and the difference between the two dilutions was used as a basis for further calculations.

In addition to quantifying the albumin recruitment, our objective was to: (1) determine whether redistributed fluid is likely to represent lymphatic flow; (2) how effectively the “washdown” expands the plasma volume, and (3) how much the process dehydrates the interstitial space.

## Materials and methods

### Evaluated trials

This retrospective study of interstitial washdown was based on the difference in plasma dilution when calculated from the blood hemoglobin (Hb) and plasma albumin concentrations during and after infusion of Ringer’s acetate in three populations (volunteers, anesthesia, and intensive care). Data from three settings were included:

1. Infusion of 25 mL/kg of Ringer’s acetate in 24 healthy male volunteers [10, 11].
2. Infusion of 25 mL/kg of Ringer’s acetate to 30 patients undergoing thyroid surgery under general anesthesia [12]. No fluid was infused during induction of the anesthesia.
3. Infusion of 1 L of 0.9% saline on 31 occasions to 17 patients (mean body weight 73 kg) with diabetes who were treated for ketoacidosis in an intensive care unit [13].

All infusions were given over 30 min.

### Procedures

The studies of volunteers and surgical patients were performed in a similar way. Both the subjects and the patients had a light breakfast consisting of one glass of water or milk and one sandwich at least 2 h before the infusion, which began at 9.00 am. They voided and were weighed just before the infusion started. The subjects rested comfortably on a bed, covered with blankets, and cannulas were inserted into the antecubital veins of both arms; one was used for infusion and the other for blood sampling. A recumbent equilibration period of 30 min was allowed before the experiments were initiated.

When an infusion had started, venous blood (3 mL) was drawn repeatedly during the infusion and for 2–3 h thereafter. The hematocrit and the blood hemoglobin (Hb) and plasma albumin concentrations were analyzed at the hospital’s clinical chemistry laboratory. Details are given the respective studies [10–13].

The diabetic patients underwent their infusion experiment soon after their arrival at the ICU. Most patients also had a repeat experiment on the next day. The choice of 0.9% saline as infusion fluid is common practice in Sweden when treating severe hyperglycemia (mean plasma glucose on arrival was 36 mmol/L) and is intended to alleviate hyponatremia and prevent cerebral edema. Here, arterial blood was sampled.

### Index of interstitial washdown

Interstitial washdown of lymph was assumed to have taken place when the recruitment of albumin to the plasma exceeded the capillary leakage of albumin. For this purpose, a comparison was made of the plasma dilution based on blood Hb vs. plasma albumin. This difference was obtained as:

1 $$\left[ {\left[ {\left. {\left( {{\text{Hb}}/{\text{hb}}} \right){-}1} \right)} \right]/\left( {1{-}{\text{Hematocrit}}} \right)} \right] - \left[ {\left. {\left( {{\text{Albumin}}/{\text{albumin}}} \right){-}1} \right)} \right]$$

Symbols in capital letters denote baseline values. For both molecules, a minor correction was made for the effects of blood sampling on the plasma dilution [12].

The difference is 0 if the recruitment of albumin to the plasma equals the capillary leakage of albumin. Positive values imply that the plasma dilution of albumin is smaller than dilution of Hb which, as Hb remains constant in the bloodstream, shows that more albumin has been added to the plasma than is lost.

The balance between albumin recruitment and capillary leakage is reported by multiplying the Hb–albumin difference in plasma dilution with the plasma albumin concentration to make the “*central albumin balance*” (unit: g/L), which then indicates the plasma albumin concentration that could be attributed to the washdown.

### Volume kinetic analysis

The central albumin balance was compared to the distribution of fluid as obtained by population volume kinetic analysis, which is based on the dilution of the Hb concentration [7]. Infusion fluids contain almost exclusively water, and the Hb dilution is therefore an index of the infused water volume that rapidly equilibrates with the site of infusion, which is the plasma. This space at baseline is given the symbol *V*_c_.

Fluid infused into *V*_c_ expands this space to *v*_c_, and the volume change is written (*v*_c_ − *V*_c_). This expansion promotes flow of fluid to a peripheral fluid space, *V*_t_, which is then expanded to *v*_t._ The flow rate (in mL/min) at any time is given by *k*_12_ (*v*_c_ − *V*_c_), which is balanced by a flow in the opposite direction occurring at the rate *k*_21_ (*v*_t_ − *V*_t_). Hence, *k*_12_ and *k*_21_ are rate constants that govern the distribution of fluid between the central (the plasma) and peripheral space (the interstitium). Elimination takes place by urinary excretion, which is proportional to (*v*_c_ − *V*_c_) by a rate constant *k*_10_.

This kinetic model, with volume exchange between two expandable body fluid compartments, is intended to reflect normal physiology. However, fluid may also accumulate in a “third space” which implies elimination from the kinetic system that is not recovered as urine. This flow represented by the rate constant *k*_b_ was called “non-exchangeable volume expansion” [2].

**Fig. 1:**
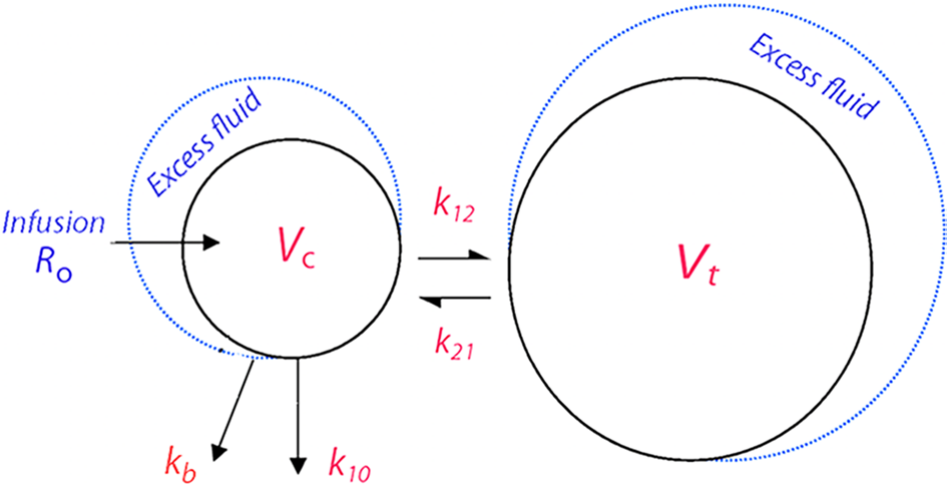
Kinetic model. Schematic drawing of the kinetic model used to analyze the distribution and elimination of Ringer’s solution

A schematic drawing of the model is shown in Fig. 1.

Differential equations for the kinetic model, in which *R*_o_ is the infusion rate, are:

2 $${\text{d}}v_{c} /{\text{dt}} = R_{o} {-}k_{12} \left( {v_{c} {-}V_{c} } \right) \, + k_{21} \left( {v_{t} {-}V_{t} } \right) {-}k_{10} \left( {v_{c} {-}V_{c} } \right) \, {-}k_{b} \left( {v_{c} {-}V_{c} } \right),$$

3 $${\text{d}}v_{t} /{\text{dt}} = k_{12} \left( {v_{c} {-}V_{c} } \right){-}k_{21} \left( {v_{t} {-}V_{t} } \right),$$

4 $${\text{d}}U/{\text{dt }} = k_{10} \left( {v_{c} {-}V_{c} } \right).$$

Dependent (input) variables were the plasma dilution, which equals (*v*_c_ − *V*_c_)/*V*_c_ in the model, and the urinary excretion (*U*), which was usually measured 2–3 times during an experiment. Equation 4 shows that the measured urine volume is used to stabilize the model by setting *k*_10_ equal to urinary excretion divided by the area under the central volume–time curve [14].

This volume kinetic model was fitted to these two dependent variables in all experiments in each cohort on a single occasion, using the Phoenix software for nonlinear mixed effects, version 1.3 (NLME, Pharsight, St. Louis, MO) and the First-Order Conditional Estimation Extended Least Squares (FOCE ELS) as search routine. Special attention was given to the modeled flows of fluid in and out of the plasma space.

The influence of interstitial washdown on the distribution and elimination of the infused Ringer’s was analyzed by using the difference in plasma dilution when based on blood Hb and plasma albumin (as written in Eq. 1) at each point in time as a potential *covariate* to each of the model parameters when Hb was used as dependent variable. The covariance was included if it reduced the residual error, expressed here as − 2(LL) (log likelihood), by > 3.8 points (*P* < 0.05). The rate constant *k*_b_, which represents “non-exchangeable volume expansion”, was included in the model if its inclusion reduced − 2LL by > 3.8 points. The linear covariate model was applied, as the difference in plasma dilution could be negative [15]. No other covariance was sought. Details about how covariance is modeled and used for simulation are given elsewhere [14].

The simulations were performed to illustrate the effect of the albumin recruitment on the distribution of infused fluid between the body fluid compartments. This was done by contrasting the bottom 5% and the top 5% differences in the Hb–albumin dilution against each other in each of the kinetic parameters for which covariance with the dilution difference was statistically significant. Graphic output was created by using MATLAB R2019b (Math Works, Inc., Natick, MA).

Demographic data were reported as the mean (standard deviation) and the kinetic data were reported as the best estimate and standard error.

## Results

### Wash-down curves

**Fig. 2:**
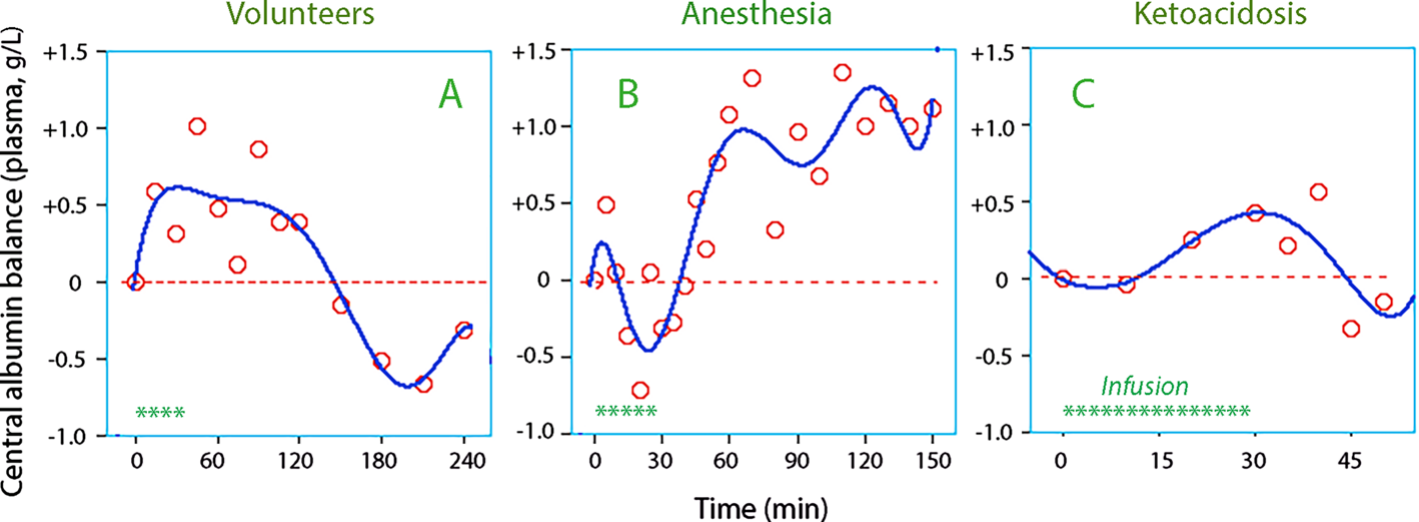
Albumin recruitment during crystalloid fluid therapy. The *y*-axis shows plasma albumin concentration that is due to interstitial washdown. Technically, each data point is the product of the Hb–albumin difference in plasma dilution and the plasma albumin concentration in **A** 20 volunteers receiving 1.7 L of Ringer’s acetate, **B** 30 patients given 1.7 L of Ringer’s acetate thyroid surgery, and **C** 31 infusions of 1 L of 0.9% saline in patients treated for diabetic ketoacidosis. Each infusion was given over 30 min

Infusion of 25 mL/kg of Ringer’s acetate solution yielded transient albumin recruitment in conscious volunteers with a maximum at the end of the 30-min infusion (Fig. 2A). Recruitment was prolonged when infused during anesthesia (Fig. 2B), but less pronounced and transient in patients with ketoacidosis (Fig. 2C). However, the ketoacidosis patients received only half as much fluid as the others.

### Volume kinetics versus washdown

**Fig. 3:**
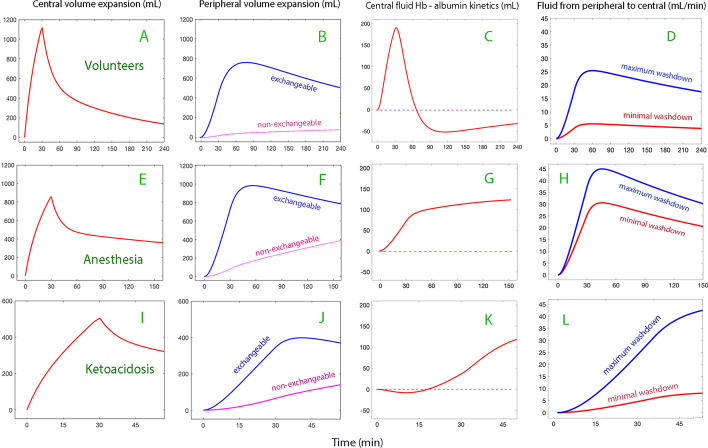
Volume kinetic analyses based on the dilution of blood Hb. The subplots in the top row show the distribution of infused fluid in volunteers between the **A** central and the **B** peripheral fluid compartment, **C** the excess fluid in the central compartment when analyzing the volume kinetics based on Hb minus the volume expansion as obtained when albumin was used as the marker of plasma dilution. **D** Return flow of fluid from the peripheral to the central space (the plasma) when contrasting the influence of high-degree versus low-degree interstitial washdown (approximately 5–95% span). Subplots **E**–**H** show the same calculations when applied to patients under general anesthesia and Subplots **I**–**L** when applied to patients with ketoacidosis

Volume kinetic analyses were used to study the prerequisites for the albumin recruitment in association with fluid therapy. Figure 3A, B shows the volume expansion of the central fluid compartment (plasma) and the peripheral compartment in the Volunteer Group. Figure 3C gives the average excess volume in the central compartment due to interstitial washdown, as given by the difference in modeled plasma volume expansion when volume kinetic analyses were based on the Hb and on the albumin dilution. The same illustrations for the other two series of infusions are shown on the subsequent rows in Fig. 3.

### Covariance with the Hb–albumin dilution difference

The volume kinetics was re-calculated using Hb as the dependent variable and with sequential examination of the Hb–albumin difference in plasma dilution at each data point (sign of albumin recruitment) as a potential covariate to the four parameters in the model. Covariance was then statistically significant in 10 of the 12 possible combinations, which confirmed that albumin recruitment affected the fluid kinetics (Table 1).

**Table 1:** Demographic data and volume kinetic analysis of the three series of crystalloid fluid infusions

	Volunteers	Anesthesia	Ketoacidosis
Demographics			
Subjects/infusions (*N*)	25/25	30/30	17/31
Females/males (*N*)	0/25	25/4	2/15
Age (years)	32 ± 7	55 ± 16	50 ± 22
Body weight (kg)	80 ± 9	69 ± 11	72 ± 11
Hb at baseline (g/L)	135 ± 8	123 ± 11*	135 ± 25
Parameter base model			
* k*_12_ (10^–3^ min^−1^)	34.0 ± 3.0	66.2 ± 7.7	83.3 ± 2.2
* k*_21_ (10^–3^ min^−1^)	12.4 ± 2.1	33.3 ± 4.5	79.4 ± 2.2
* k*_10_ (10^–3^ min^−1^)	11.8 ± 0.2	2.4 ± 0.5	13.2 ± 2.6
* k*_b_ (10^–3^ min^−1^)	1.1 ± 0.1	5.4 ± 1.6	5.8 ± 2.6
Covariance			
* k*_12_	6.7 ± 1.9	2.3 ± 0.7	None
* k*_21_	7.3 ± 4.9	2.7 ± 1.2	-0.9 ± 0.1
* k*_10_	− 4.2 ± 0.9	2.9 ± 1.4	-1.7 ± 0.8
* k*_b_	None	5.2 ± 2.8	-2.3 ± 0.3

The fluid distribution is given by the four top rate constants. These are modified by covariance with the difference between the Hb and albumin dilution, representing the washdown of interstitial albumin, at each point in time in each subject (time-varying covariate). Linear covariance models were used

Data are the mean ± SD. *After induction of general anesthesia

This covariance analysis was used to create plots that contrast minimal albumin recruitment (lowest 5%) from pronounced albumin recruitment (highest 5%) from each other. Figure 3D shows how washdown affected the flow of fluid from the peripheral to the central fluid space.

**Fig. 4:**
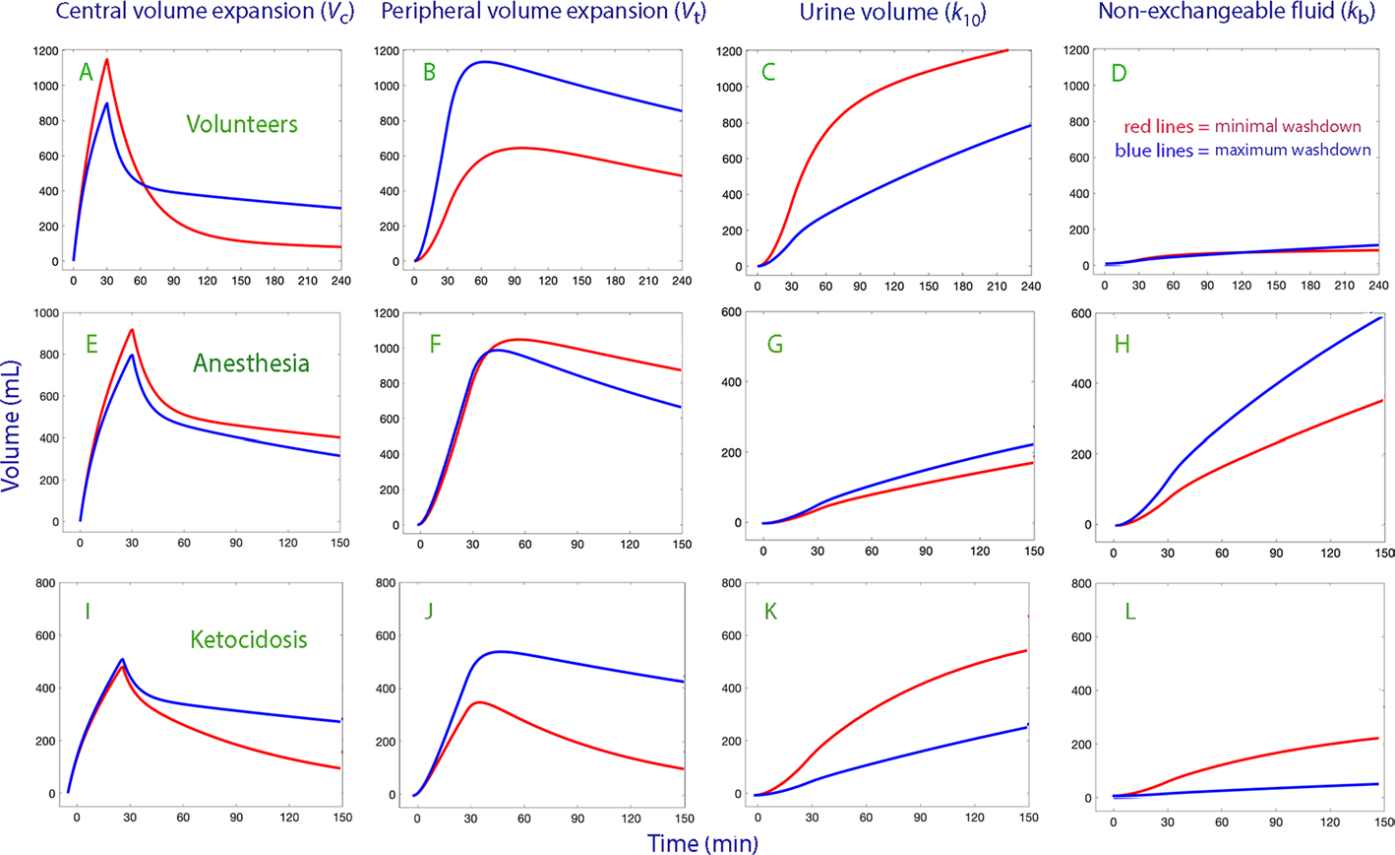
Influence of interstitial washdown on the distribution of crystalloid fluid. Volume kinetic analysis of the fluid distribution when 1.7 L of Ringer’s was infused in volunteers (top row; subplots **A**–**D**), 1.7 L to patients undergoing surgery (middle row; subplots **E**–**H**) and 1.0 L of 0.9% saline was given to patients with ketoacidosis (bottom row; subplots **I**–**L**). All infusions were given over 30 min. All volumes are shown depending on whether the interstitial washdown was in the low or high range (minimal to maximum washdown; approximately 5–95% span). For the volunteers, the range was between − 0.10 and + 0.10 (mean − 0.019), for the anesthesia patients − 0.01 to + 0.15 (mean, + 0.05), and for the patients with ketoacidosis the Hb–albumin difference in plasma dilution varied from − 0.10 to + 0.40 (mean, + 0.046)

Figure 4 illustrates how the washdown affected the distribution of infused Ringer’s between all modeled body fluid spaces.

### Contribution of lymph

The build-up of excess plasma volume due to interstitial washdown required approximately 30 min in all three experiments (Fig. 3C, G, K). Calculations were performed to examine to what degree the modeled inflow of fluid to the plasma consisted of lymph.

A turnover equation was used to estimate the albumin concentration of the inflow during this 30-min build-up period. The equation holds that the recruited amount of albumin per minute equals the input minus the elimination during the same time period. Capillary leakage of albumin in healthy volunteers is considered to occur at a rate of 5% of the intravascular pool per hour in volunteers [16], which corresponds to a clearance (CL) of 3.5 mL/min if the plasma volume is 3 L.

For the Volunteer Group, the increase in central albumin difference was 0.5 g/L (Fig. 2A), the plasma volume approximately 3 L, the average inflow of fluid 6 mL/min between 0 and 30 min, and plasma albumin 36 g/L. Hence, for 30 min:

5 $$0.5 \times 3 \times 10^{3} = 6 \times 30 \times X{-}3.5 \times 36 \times 30;\,X = 29{\text{ g}}/{\text{L}}.$$

In the Anesthesia Group, inflow averaged 11 mL/min and washdown increased plasma albumin by 1.0 g/L (Fig. 2B). On the assumption that CL was doubled during surgery [17], the albumin concentration of the inflow was estimated to be 29 g/L, i.e., the same as in the volunteers. The same calculation for the Ketoacidosis Group yielded 22 g/L.

During the period of steady state, the inflow of albumin (inflow rate × *X*) equals the outflow (CL × plasma albumin) [18]. The volume kinetic analysis showed that the inflow rates during these periods averaged 9, 28, and 31 mL/min while the measured plasma albumin averaged 36, 31, and 31 g/L for the three study groups, respectively. The albumin concentrations of the recruited fluid so obtained were 14, 8, and 7 g/L. Hence, the albumin concentration in the accelerated inflow decreased over time.

### Lymph flow at baseline

The baseline lymph flow is not included in the calculations presented above. However, the baseline inflow before the Ringer infusion can be estimated by knowing that the albumin concentration in lymph is normally 50–70% that of the plasma [8]. Plasma albumin at steady state (*C*_*ss*_) then equals the albumin inflow divided by the CL. This means that the baseline lymph flow will be twice the CL, i.e., 7 mL/min, regardless of plasma albumin concentration. If all fluid flowing into the plasma from the interstitium consists of lymph, this calculation implies that the lymph flow initially increased by 3–4 times in response to crystalloid fluid loading.

## Discussion

### Physiological basis of washdown

Crystalloid fluid leaves the vascular system with a distribution half-life of only 8 min [7] leading some to question their utility in fluid resuscitation [19]. In the present subjects, fluid loading caused a five- to sixfold increase in capillary filtration. Such rapid leakage of crystalloid into the interstitial space probably drives an increase in lymph flow resulting in a decrease in interstitial protein concentration [3]. This might occur because the transendothelial leakage of protein occurs more slowly than the return of proteins from the lymphatic system to the plasma when the lymphatic flow is accelerated. Hence, interstitial protein “washdown” should be considered to be a safety factor against tissue edema [3]. The protein concentration of the plasma would increase under such circumstances, and this could act to improve the plasma volume expansion induced by the infused fluid.

We undertook an analytical approach to plasma albumin refill by assessing kinetics in multiple compartments to characterize such interstitial washdown in living humans. This approach has never been applied to albumin refill and the results present novel findings that provide evidence as to the mechanism(s) of albumin refill that have previously evaded physiologists and clinicians.

### Key results

Our present results show that, by infusing 1–2 L of crystalloid fluid in humans, interstitial washdown increased plasma albumin by 0.3–1.0 g/L. The increase in plasma albumin occurred over approximately 30 min, which was also the infusion time. However, the kinetic analysis suggests that overall effect of the washdown on the fluid distribution was far greater than accounted for by the oncotic properties of the recruited albumin.

The results further agree with the view that the recruited albumin is derived from lymph. The evidence stems from our estimates of the albumin concentration in the inflowing fluid during the build-up phase, which was the same as the 29 g/L in interstitial fluid of the forearm of adult males measured by the wick method [9]. However, the concentration decreased over time, suggesting that washdown of albumin becomes exhausted after a couple of hours. Similarly, the protein concentration of collected lymph of instrumented dogs had dropped by 50% after 2 h in response to three 5-min bolus infusions of saline [6].

These results also imply that the rate constant *k*_21_ in volume kinetics represents lymphatic flow when crystalloid fluid is infused.

### Simulated effects of washdown

The modifying influences of interstitial washdown on the fluid distribution in our studied settings were highlighted by simulations (Fig. 4). They do not appear to be negligible. Pronounced washdown in the volunteers was associated with greater central and peripheral volume expansion. The plasma volume expansion was clearly prolonged at the expense of the urinary excretion, which was reduced by 1/3. Surprisingly, no protection from peripheral edema was apparent. However, the covariance analysis showed that pronounced interstitial washout is associated with a very high *k*_12_, which means that the peripheral accumulation of fluid could have been even greater without the washdown.

During general anesthesia the main effect of the washdown was an increase of the non-exchangeable (“third space”) volume expansion, which is known to occur when urinary excretion is restricted despite adequate body hydration [2]. This finding merits further study, as non-exchangeable fluid might remain in the body for a long time. The influence of washdown on the expansion of the plasma and the interstitial volume was almost negligible general anesthesia.

By contrast, the simulated effects of interstitial washdown in the diabetic patients showed a pattern similar to the one described for healthy volunteers.

Little enhancement of the plasma volume expansion could be discerned during the acute build-up phase of albumin enrichment, but the plasma volume expansion was twice as large as 3-h post-infusion in conscious subjects who had pronounced washdown than in the others. This late effect is not apparent in the Anesthesia Group and is probably due mostly to the inhibitory effect of interstitial washout on urinary excretion.

### Pathological states

Studying fluid infusion on albumin refill has widespread clinical importance. Rapid fluid infusion is a first line strategy to treat hemorrhage, hypovolemia, and changes in vascular capacitance such as sepsis and following the induction of general anesthesia. The increased filtered fluid volume following rapid infusions of crystalloid solution must be returned to the plasma to maintain a healthy balance between the extravascular compartments. However, there is evidence to suggest that the balance may not always be healthy.

Animal studies of sepsis [20] and the transurethral resection syndrome [21] demonstrate that *k*_21_ is strongly reduced or even be zero in these shock states, whereby interstitial washdown is likely to have ceased. Other experimental studies give clues to why the washdown mechanism is weakened or even disappears. Sepsis acutely decreases the interstitial pressure by several multiples due to protease-mediated cleavage of interstitial matrix elements, which increases capillary filtration and promotes interstitial accumulation of fluid and albumin [22]. Moreover, TNF-alpha, prostaglandins, and nitric oxide (NO) inhibit lymphatic pumping as the inflammatory process continues, which might even lead to lymphatic pump failure [23, 24].

Lack of return of filtered fluid and albumin in the form of interstitial washdown to the plasma would promote the development of hypovolemia, extravascular edema, and hypoalbuminemia.

### Is lymph recruited?

The albumin concentration in the early inflow averaged 70–80% of the baseline plasma albumin, which supports that lymph was recruited. Similar ratios have been obtained in volunteers [9] and dogs [6] while being only 50% in rabbits [8]. The alternative route for the recruitment would be across the capillary endothelium and the glycocalyx, but the fluid and the albumin would then have to pass at the same rate as recruited fluid had an albumin concentration that was identical to, or very close to, the lymph.

We hypothesize that the albumin concentration of the inflow is not regulated, but is a summation of the hydration status and the capillary filtration of fluid and albumin. Increased capillary leakage of albumin would probably increase of albumin concentration of the inflowing fluid while persistently increased fluid filtration would dilute the albumin concentration. The process will eventually subside as the amount of available albumin in the interstitial space decreases.

The thoracic duct has often been considered as the main route of albumin recruitment. Thoracic duct lymph albumin concentration shows no change or actually decreases in response to blood loss, which has been explained as a selective retention of albumin within the circulation. However, thoracic duct albumin increases when hemorrhage is treated by rapid infusion of lactated Ringer’s, which might be explained by increased filtration and subsequent acceleration of the lymphatic flow. These results only account for changes in visceral lymph and do not represent what happens in the total body [25]. Our approach provides an analysis of whole-body albumin refill which provides a more complete characterization.

### Effects not due to recruited albumin

Translocation of fluid to the plasma likely occurs due to the oncotic properties of recruited albumin. However, as each gram of albumin binds approximately 10–11 mL of fluid [26, 27] which, with a plasma volume of 3 L, accounts for only 17 mL in the Volunteer Group and twice as much in the Anesthesia Group. Our calculations then imply that interstitial washdown changes the fluid distribution much more greater than can explained by albumin alone.

Four factors may contribute to this discrepancy. First, lymphatic immunoglobulins with oncotic properties accompany the translocated albumin. Second, the concentrations of albumin and immunoglobulins in the interstitium, with which the plasma is at balance, decrease. Third, the inhibitory effect of the interstitial washdown on the urinary excretion increases both the plasma and the interstitial fluid volumes. Fourth, some of the redistribution shown in Fig. 4 might still be due to hydrostatic and viscosity consequences of the high *k*_12_ values associated with albumin recruitment.

### Capillary refill

Capillary refill occurs in response to hypovolemia and, as demonstrated here, to crystalloid fluid administration. There are two distinct components: the first one is the translocation of fluid and the second the return of protein, specifically albumin, into the plasma. This indicates that albumin refill occurs as part of fluid refill and, in fact, might be crucial for maintaining the process [28].

The source and mechanism(s) of albumin refill have been a matter of controversy for over 40 years [25, 29, 30], but may have important physiological and therapeutic consequences. Francis D. Moore and colleagues were the first to comment that while transcapillary refill results in marked hemoglobin dilution while plasma albumin remains constant, suggesting that albumin is returned to the vascular compartment at a rate proportionately greater than fluid [30–34]. However, albumin enrichment in the plasma can occur despite slow recruitment of albumin because fluid leaks out 75 times faster from the plasma than albumin does.

Fogh-Andersen et al*.* [35] estimated that the interstitial albumin concentration averaged one-third of the plasma concentration by comparing the difference in Hb and albumin dilution 10 min after volunteers had changed body position from standing to supine, which is known to rapidly recruit fluid [5]. This approach is similar to “central albumin balance” we used. The result emphasizes the high speed of the albumin recruitment, although the lymph was not implied as being the source.

### Limitations

Certain known differences in the kinetics of crystalloid fluid between the three clinical settings has previously been described and should be considered when reviewing the effects of interstitial washdown. The most important difference is that the diuretic response to volume loading is strong in volunteers but much weaker (− 90%) during general anesthesia [14]. “Non-exchangeable” volume expansion is pronounced during general anesthesia [2], intermediate in diabetic ketoacidosis [13], and very small or even absent in healthy volunteers [7].

Other limitations include that the data used for the analyses stem from previously published works that were performed for other purposes. The mass balance calculations report only mean data because the differences in dilution between Hb and albumin were so small that inter-individual variability would be strongly affected by measurement precision. Albumin clearance values were taken from the literature.

The volume kinetic analysis is likely to closely capture real physiological events [36], but uncertainties exist about the nature of the non-exchangeable volume expansion [2]. This fluid remains in the body but without equilibrating with the plasma within the 3–4 h of the experiment. During anesthesia and surgery one-third of the infused crystalloid fluid is at least temporarily unavailable for redistribution and excretion, which probably contributes to postoperative weight increase and edema. However, further exploration of the “third spacing” phenomenon is beyond the scope of the present work.

Benefits with our approach include minimal invasiveness and that our model for studying albumin refill is devoid of the many sympathetically mediated compensatory responses induced by hemorrhage [4].

## Conclusion

Our novel analytical approach demonstrates that crystalloid volume loading induces capillary refill of both fluid and albumin. The increase of plasma albumin is modest while the plasma volume expansion becomes prolonged in conscious subjects but not in anesthetized patients. The albumin concentration of the recruited fluid is similar to the interstitial fluid early on while a reduction by 50–75% has occurred after 1–2 h. These data contribute to an enhanced understanding of the physiological effects of fluid administration on plasma volume expansion and dispel long held thinking about fluid and peripheral edema.

## Data Availability

The original data are available upon request from the RGH.
